# Proximal tubule cells in blood and urine as potential biomarkers for kidney disease biopsy

**DOI:** 10.7717/peerj.16499

**Published:** 2023-12-06

**Authors:** Minwa Lin, Yingxue Zhong, Dan Zhou, Baozhang Guan, Bo Hu, Panpan Wang, Fanna Liu

**Affiliations:** 1 Depament of Nephrology, The First People’s Hospital of Foshan, Foshan, China; 2 Depament of Nephrology, The First Affiliated Hospital of Jinan University, Guangzhou, China; 3 Cancer Center, The First People’s Hospital of Foshan, Foshan, China; 4 Department of Traditional Chinese Medicine, the First Affiliated Hospital of Jinan University, Guangzhou, China

**Keywords:** Nephritis, Neutropil, Inflammatory proximal tubule cells, Cell exfoliation, Single-cell sequencing

## Abstract

Early diagnosis and treatment are crucial for managing kidney disease, yet there remains a need to further explore pathological mechanisms and develop minimally invasive diagnostic methods. In this study, we employed single-cell RNA sequencing (scRNA-seq) to assess the cellular heterogeneity of kidney diseases. We analyzed gene expression profiles from renal tissue, peripheral blood mononuclear cells (PBMCs), and urine of four patients with nephritis. Our findings identified 12 distinct cell subsets in renal tissues and leukocytes. These subsets encompassed fibroblast cells, mesangial cells, epithelial cells, proximal tubule cells (PTCs), and six immune cell types: CD8+ T cells, macrophages, natural killer cells, dendritic cells, B cells, and neutrophils. Interestingly, PTCs were present in both PBMCs and urine samples but absent in healthy blood samples. Furthermore, several populations of fibroblast cells, mesangial cells, and PTCs exhibited pro-inflammatory or pro-apoptotic behaviors. Our gene expression analysis highlighted the critical role of inflammatory PTCs and fibroblasts in nephritis development and progression. These cells showed high expression of pro-inflammatory genes, which could have chemotactic and activating effect on neutrophils. This was substantiated by the widespread in these cells. Notably, the gene expression profiles of inflammatory PTCs in PBMCs, urine, and kidney tissues had high similarity. This suggests that PTCs in urine and PBMCs hold significant potential as alternative markers to invasive kidney biopsies.

## Introduction

The kidney is a highly complex and organized organ, made up of more than 40 distinct cell types (Lindstrom et al., 2018). Its main filtering unit, the glomerulus, plays a pivotal role in maintaining the body’s fluid homeostasis, which includes the conservation of water and the balance of acids, bases, and electrolytes. Several factors such as drugs, infections, idiopathic conditions, and systemic inflammation can trigger renal diseases such as glomerulosclerosis and diabetic nephropathy (DN). These conditions, in turn, can progress to chronic kidney disease (CKD).

Early diagnosis and intervention can significantly enhance the prognosis of kidney diseases. The prevailing diagnostic approaches for kidney diseases predominantly rely on imaging techniques (ultrasound and magnetic resonance imaging) and biomarkers including creatinine, cystatin C, albuminuria, estimated glomerular filtration rate, urea, and uric acid. Nonetheless, the limitations in sensitivity and specificity of these techniques hinder their ability to detect early-stage diseases, potentially resulting in delayed treatments (Wong & Pollock, 2014). While histopathology can offer precise insights into kidney disease progression, it requires an invasive renal biopsy, making it unsuitable for routine prognosis assessments.

Transcriptomic analysis, which centers on changes in gene expression, presents promising prospects in the realm of kidney disease research. It not only provides disease classification and prognostic data (Liu et al., 2022) but also sheds light on the underlying mechanisms of pathogenesis, subsequently guiding therapeutic interventions for disease management (Eddy, Mariani & Kretzler, 2020; Grayson et al., 2018; Kingsmore et al., 2021). However, a shortcoming of bulk transcriptome profiling is its propensity to predominantly capture data from major cell populations. Consequently, anomalies in a few functional renal cells might be overshadowed by the overwhelming majority of normal cells (Wu & Humphreys, 2017). scRNA-Seq, a technique widely adopted for single-cell transcriptome analyses, has revolutionized our understanding of diverse cell types in tissues, highlighting differences between cells, especially the rarer ones. Crucially, it can delineate the developmental pathways of cells associated with disease causation. This unbiased single-cell sequencing defines kidney cells by their transcriptomes, facilitating the identification of changes in rare cell expression profiles and cell–cell interactions during disease progression (England et al., 2020; Park et al., 2018; Zambrano et al., 2022; Zhu, Lu & Weng, 2023). This in-depth transcriptional profiling at the cellular level has significantly enhanced our understanding and management of kidney diseases (Chen et al., 2021; Heintze, 2018; Peng et al., 2023; Schena et al., 2021; Zeng et al., 2021).

Addressing the pressing need for early and accurate renal disease diagnoses has become a major global public health priority. Current diagnostic markers, such as proteinuria and serum creatinine, are influenced by patients’ physiological conditions. Often, alterations in these indicators do not align timely with renal damage. Renal biopsy, although considered the gold standard for diagnosing kidney diseases, is invasive and may miss the identification of early disease indicators. In this study, scRNA-seq was employed to analyze cells from blood and urine. The goal was to elucidate the pathological development of renal diseases and to identify potential novel biomarkers. This could pave the way for alternative minimally invasive or even non-invasive diagnostic methods for kidney diseases.

## Material and Methods

### Sample acquisition

Renal tissue, urine, and blood specimens were collected from four patients diagnosed with membranous nephropathy (MN), interstitial nephritis (IN), proliferative and sclerosing glomerulonephritis (PSG), and DN. These diagnoses were confirmed through pathological examination of renal needle biopsies. All participating patients provided written informed consent. The study received approval from the Institutional Ethics Board of the First Affiliated Hospital of Jinan University (approval no. KY-2020-034). Biopsy samples were collected from subjects who had provided informed consent, and the samples were obtained during a clinically indicated biopsy procedure, and paired blood and urine samples were concurrently collected. After the biopsy, the excised kidney specimens were transported to a technical laboratory on ice within 2 h.

Single-cell suspensions from tissue were prepared as previously described, with some modifications (Park et al., 2018). Briefly, the renal biopsy samples were washed with ice-cold Roswell Park Memorial Institute medium, minced into approximately 1 mm^3^ granules, and then digested using the Multi Tissue Dissociation kit (Miltenyi, Bergisch Gladbach, Germany). This digestion occurred at 37 °C for 30 min and was halted by adding fetal bovine serum to achieve a 10% final concentration. The resultant cell suspension was sieved through a 40-µm filter, and cells were gathered by centrifuging at 1,000 rpm for 5 min. Cell counts and viability were assessed using Trypan blue staining, revealing a viability range of 60%–80%. Blood cells were collected by centrifugation for 5 min at 1,000 rpm and 4 °C, followed by erythrocyte lysis with one mL of red blood cell lysis buffer on ice for 3 min. Similarly, urine cells were collected by centrifugation for 5 min at 1,000 rpm and 4 °C. All isolated cells were subjected to immediate single-cell sequencing analysis.

### Single-cell sequencing

Single-cell RNA sequencing (scRNA-Seq) libraries were crafted using 10X genomics kits according to the manufacturer’s instructions (Park et al., 2018). Briefly, single-cell beads in emulsion (GEMs) were created using the Chromium single-cell controller (10 × Genomics) with a concentration of 300-600 viable cells/mL. RNA underwent barcoding through reverse transcription at 53 °C for 45 min after cell lysis. The reaction was then terminated at 85 °C for 5 min. Barcoded cDNA was amplified with 35 cycles of PCR amplification(95 °C for 30 s; 95 °C for 20 s, 67 °C for 30 s, 72 °C for 1 min). scRNA-seq libraries were constructed using a single-cell 5′ library according to the manufacturer’s instructions. scRNA-Seq data of healthy blood were sourced from the Gene Expression Omnibus at NCBI (GSE157278) and underwent identical analytical procedures.

### scRNA-Seq data processing and quality control

Sequencing data was analyzed using the BD Rhapsody analysis pipeline. Gene identity was identified by referring to the hg19 version of Gencode, with further analysis using the Seurat R package. Quality control was conducted by removing log-library that has more than three median absolute deviations (MADs) below the median log-library and log-genes outliers based on a threshold of 3 MADs from the median. Cells with more than 20% mitochondrial genes were also discarded.

### Unsupervised clustering and marker identification

Seurat R package (v3.2.3) facilitated the unsupervised clustering of single cells (Stuart et al., 2019). Genes detected in fewer than 10 cells were excluded from subsequent analyses. The principal component analysis focused on approximately 2000 highly variable genes. The FindClusters function, with a resolution of 0.4, was used to perform the cell clustering. Differentially expressed genes (DEGs) in more than 30% of cells across clusters were identified through the Wilcoxon rank-sum test.

### Dimensionality reduction using t-SNE

Visualization of cell distances in a reduced 2D space was performed by using the Barnes-Hut t-distributed stochastic neighbor embedding (t-SNE) method. The Seurat v3 Integrate Data function assisted in the integrated analysis (CCA).

### Gene set variation analysis

The gene set variation analysis package (version 1.3.0) executed the gene set enrichment analysis (GSEA), with results exported using the GSEABase package (version 1.44.0). Differences in per-cell pathway activities were analyzed with the LIMMA package (version 3.37.11). The identified DEGs with a fold change of ≥2 or ≤0.5 were categorized, followed by clustering based on each gene’s area-under-the-curve value. Clusters were identified by analyzing their gene expression and were named according to their top 1,000 signature genes. The sequencing data are available on GEO: GSE203406.

### Pathway analysis

For pathway enrichment analysis, genes with a Benjamini–Hochberg-adjusted *P*-value < 0.05 and an absolute log2 (fold change) greater than 0.25 were analyzed with DAVID pathway enrichment analysis (https://david-d.ncifcrf.gov/). GSEA was also performed. The signal-to-noise ratio, derived from dividing the log2 FC by the standard deviation, ranked the genes.

## Results

### Twelve cell clusters were identified in the tissue sample

**Figure 1:**
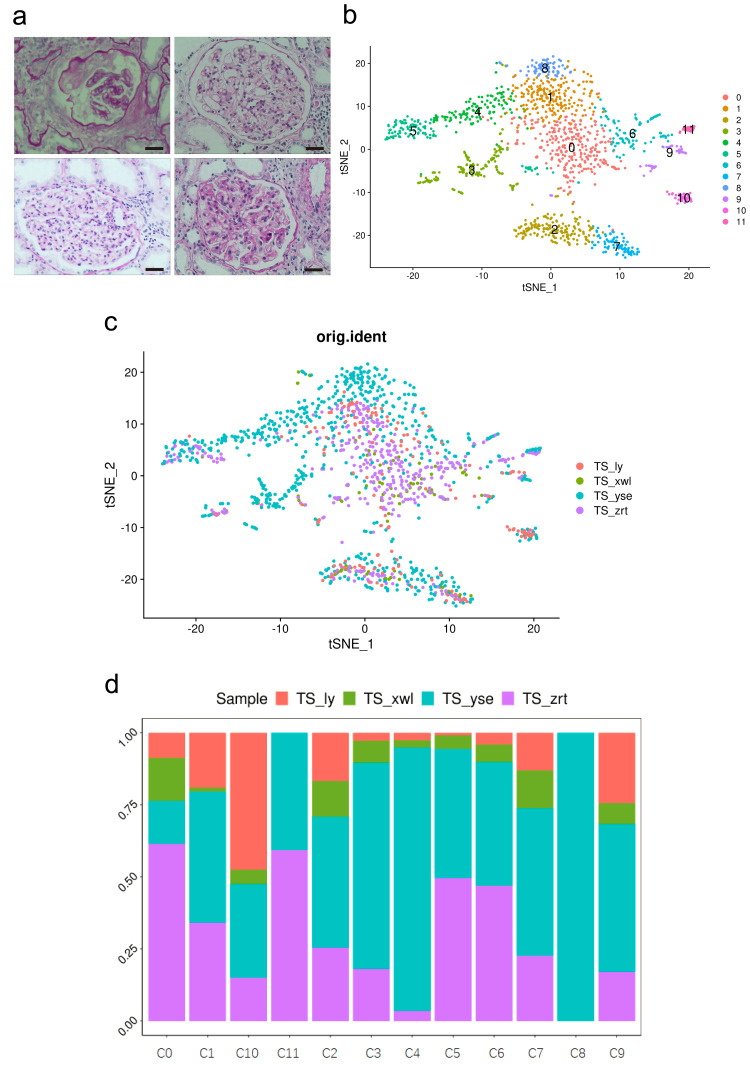
Pathological changes and global renal cell atlas of renal tissues. (A) Hematein-eosin staining of renal tissues collected from the four patients. Scale bar = 20 µM. (B) t-Distributed stochastic neighbor embedding showing the 12 distinct cell types. Cluster 0, fibrotic fibroblast cells. Cluster 1, preapoptotic mesangial cells. Cluster 2, CD8 + T cells. Cluster 3, epithelial cells (descending and ascending limbs). Cluster 4, inflammatory proximal tubule cells. Cluster 5, proximal tubule cells. Cluster 6, macrophages. Cluster 7, natural killer cells. Cluster 8, fibroblasts. Cluster 9, dendritic cells. Cluster 10, B cells. Cluster 11, neutrophils. (C) Distribution of cells from the four samples in the 12 clusters. (D) Distribution of cells from the four samples in the 12 clusters.

Peripheral blood mononuclear cells (PBMCs) were collected from four patients confirmed with MN, IN, PSG, and DN through histological examination (Raw data available in Files S1), as well as through blood routine and biochemical tests. The clinical characteristics and laboratory examination data of the patients are outlined in Table S1. Pathology images of the four patients are displayed in Fig. 1A. A total of 1,369 cells from biopsies, 12,815 cells from blood samples, and 6,278 cells from urine samples successfully passed the quality control. Clusters were annotated using canonical markers as previously characterized (Lake et al., 2019).

Cells within the renal tissue can be categorized into 12 distinct clusters (Fig. 1B), comprising fibroblast cells (pro-fibrosis, F-FC), mesangial cells (pro-apoptotic, P-MC), epithelial cells (descending and ascending limb, EC), proximal tubule cells (PTCs), inflammatory PTCs (inflammatory PTCs and Infla-PTCs), fibroblast cells (FCs), CD8+ T cells, macrophages (MCs), natural killer cells (NKs), dendritic cells (DCs), B cells, and neutrophils. Ten clusters encompassed cells from samples of the four different patients (Fig. 1C). Cluster 8 (FC) exclusively included cells from one patient, while cluster 11 (neutrophils) encompassed cells from two patients. The distribution of cells from the four samples across the 12 clusters is detailed in Fig. 1D (Table S2).

**Figure 2:**
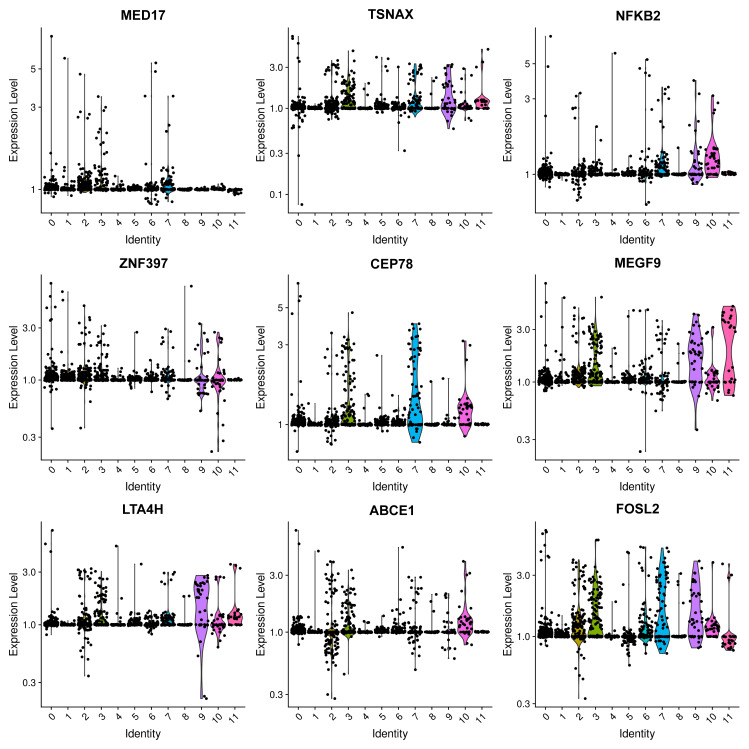
Violin plots showing the expression levels of representative marker genes of Cluster 0 across the 12 clusters.

The most prevalent clusters are the fibroblast cells, including cluster 0 and cluster 8. Cluster 0 exhibits a high expression of leukotriene A4 hydrolase (LTA4H) (Fig. 2), which is responsible for the production of LTB4 (Wan et al., 2017). LTB4 can lead to an increased influx of neutrophils and T cells; however, sustained LTB4 production may induce sterile inflammation (Subramanian, Majumdar & Parent, 2017). Normal fibroblast cells were only observed in patient #3 with chronic IN, aligning with the negative staining of IgG, IgM, IgA, C3, and C1q. The remaining three patients displayed positive staining for at least one antibody or complement. Consequently, the elevated expression of LTB4 in the samples from all four patients suggests that LTB4 could potentially serve as a marker for MN, IN, PSG, and DN.

**Figure 3:**
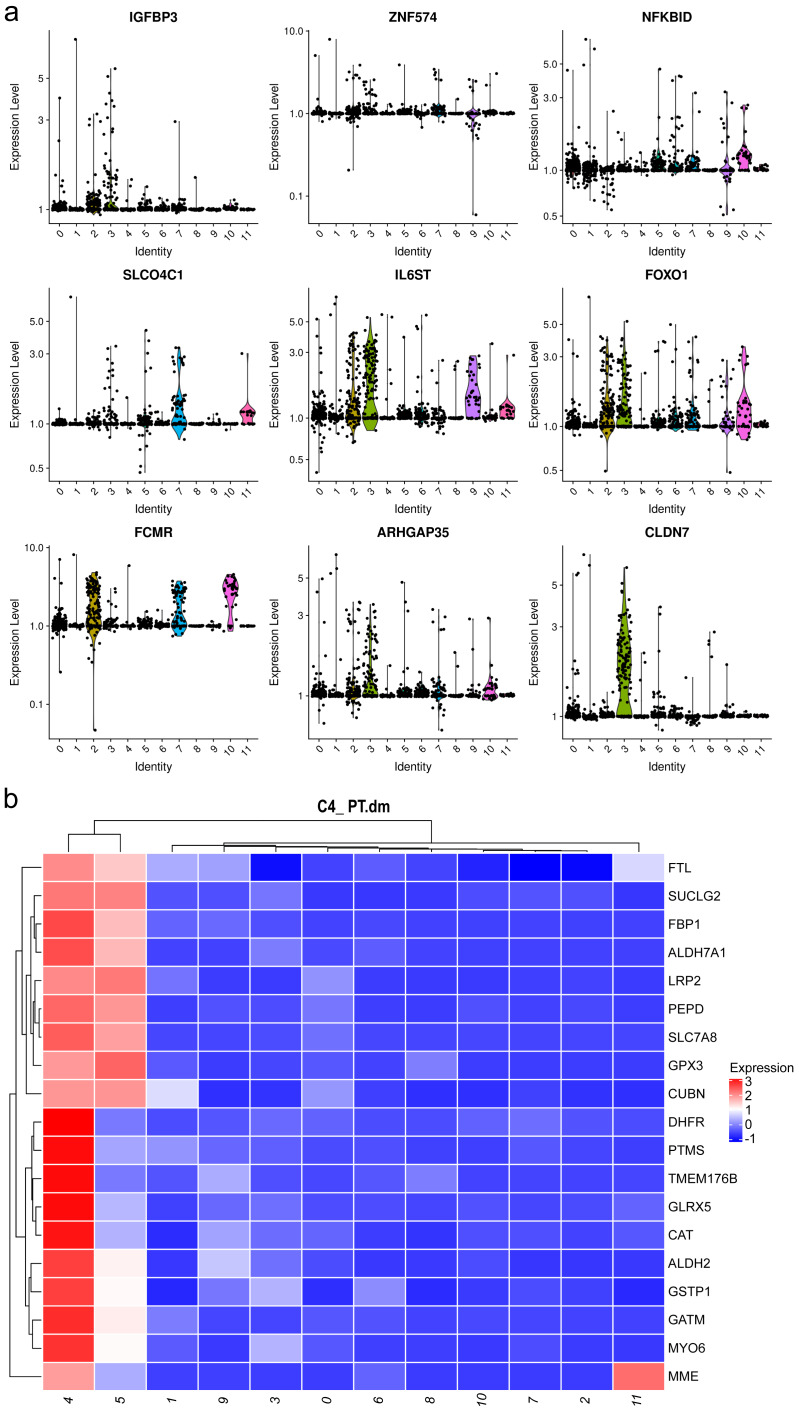
Violin plots showing the expression of representative marker genes of Cluster 1 across the 12 clusters.

Mesangial cells constitute the second most abundant clusters. Among the top 20 markers for cluster 1, two mitochondrial genes were identified (Fig. 3A, Table S3), implying an association with energy-intensive molecule transportation or reabsorption (Ilicic et al., 2016). Generally, mesangial cells are characterized by low mitochondrial gene expression. Elevated levels of mitochondrial genes are typically found in healthy renal cells, such as proximal tubules (PTs) and thin ascending limbs (Lake et al., 2019). Consequently, we examined other top genes and identified the pro-apoptotic gene IGFBP3. IGFBP 3 mediates apoptosis of mesangial cells (Vasylyeva, Chen & Ferry Jr, 2005) and is elevated in patients with CKD (Buscher et al., 2012). Considering the pathological background of the patients, the heightened levels of mitochondrial genes and IGFBP 3 suggest a preapoptotic state of these mesangial cells.

Both clusters 4 and 5 were identified as PTCs. Cluster 4 displayed a notable elevation in catalase, ALDH2, and Gstp1(Fig. 3B). Elevated intracellular levels of reactive oxygen species (ROS) play a major role in the pathogenesis of CKD. The increased expression of catalase and ALDH2 could counterbalance ROS-mediated injury (Chen et al., 2015; Irazabal & Torres, 2020). Gstp1 also plays an important role in detoxification with reduced glutathione, which was also upregulated in glomerulosclerosis (Miyazaki et al., 2014). Thus, these cells are in a ROS-mediated inflammation state. Therefore, cluster 4 was categorized as inflammatory PTC.

Six types of immune cells were identified in the sample (Fig. 1B), including CD8+ T cells (cluster 2), MC (cluster 6), NK cells (cluster 7), DCs (cluster 9), B cells (cluster 10), and neutrophils (cluster 11). This cellular pattern, excluding neutrophils, has been previously reported in the kidneys of patients with lupus nephritis (Arazi et al., 2019). Neutrophils are commonly found in renal tissues affected by neutrophil-mediated kidney inflammation. However, scRNA-seq analysis of healthy kidney tissue indicates that only tissue-resident macrophages are present in the kidney tissue from tumor-free regions of nephrectomies (Lake et al., 2019). Moreover, no B cells were observed in healthy kidney tissues (Arazi et al., 2019). These findings underscore the pathological alterations observed in these patients.

### Classification and annotation of PTCs in blood and urine samples

**Figure 4:**
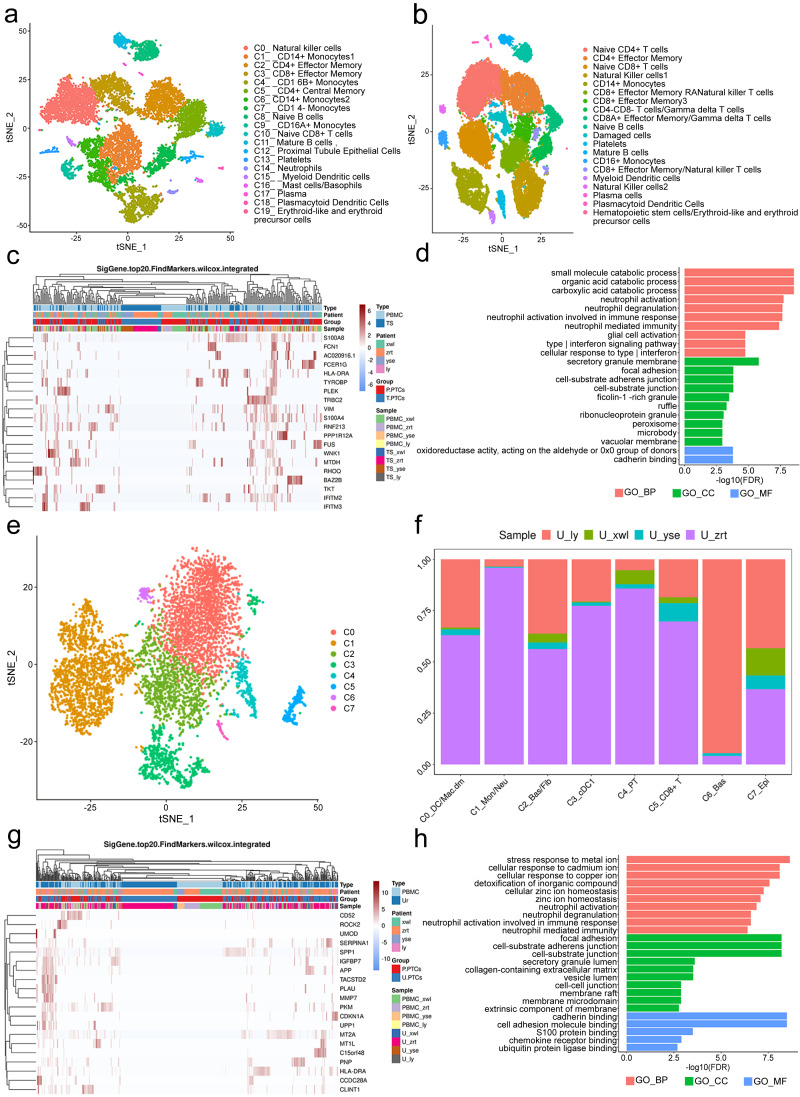
Classification of proximal tubule cells (PTCs) and gene ontology (GO) analysis of differentially expressed genes (DEGs) in blood and urine PTCs. (A) t-Distributed stochastic neighbor embedding clustering of blood cells from the four patients. (B) t-Distributed stochastic neighbor embedding clustering of blood cells from the five healthy control subjects. (C) Top 20 DEGs of PTCs in peripheral blood mononuclear cells (PBMCs) and tissues. (D) Top 20 significant enriched pathways of DEGs in PTCs between PBMCs and tissues. (E) t-Distributed stochastic neighbor embedding clustering of urine cells from the four patients. (F) Distribution of urine cells from the four samples in the seven clusters. (G) Top 20 DEGs of PTCs in PBMCs and urine. (H) Top 20 significant enriched GO pathways of DEGs in PTCs between PBMCs and urine.

A total of 12,815 blood cells were analyzed and classified into 19 clusters, predominantly comprising immune cells (Fig. 4A). It is worth noting the presence of PT epithelial cells (cluster 12) in the blood, accounting for 1.58% of the total cells. No PTCs were detected in peripheral blood from the five healthy control subjects (Fig. 4B). There is also no documented evidence of PTCs in peripheral blood, suggesting their potential utility as a blood-based biomarker for nephritis diagnosis.

DEGs in PTCs from PBMCs and tissues were further scrutinized. A total of 558 DEGs were identified, encompassing 556 genes with upregulated expression and two genes with downregulated expression (Table S4) in PTCs from PBMCs. Notably, the top 20 DEGs included vimentin (VIM) and interferon-induced transmembrane proteins 2 and 3 (IFITM2 and IFITM3) (Fig. 4C). VIM encodes intermediate filament proteins that regulate the dynamics of cell fiber networks. Upregulated expression of vimentin not only enhances cell motility in epithelial cells but also results in the loss of cell–cell contacts and increased turnover of focal adhesions (Mendez, Kojima & Goldman, 2010; Rogel et al., 2011). Given that PTCs exfoliate from the tissue, significant changes in cell shape occur, justifying the upregulation of vimentin in circulating PTCs. This finding aligns with the Gene Ontology (GO) analysis, which highlights the involvement of neutrophil and interferon-related pathways, as well as components related to cell–cell interactions in nephritis development (Fig. 4D). Additionally, IFITM2 and IFITM3 were also upregulated in circulating PTCs. These proteins restrict virus infections by blocking membrane fusion. High expression of IFITM2 and IFITM3 has been widely detected in individuals with COVID-19 (Ramos et al., 2020). In the absence of infection in the four patients, it is conceivable that IFITM2 and IFITM3 are implicated in aseptic inflammation and cell detachment of PTCs. Given that many of the top GO terms are related to inflammation and cell adhesion, uncontrolled inflammation or injury may induce a preapoptotic state in PTCs, ultimately leading to their exfoliation from the tissue.

Viable cells in the urine sample were significantly fewer than those in tissue or blood, with 6,278 cells passing the quality control and classified into seven clusters. These included apoptotic plasma cells (cluster 0), neutrophils (cluster 1), fibroblasts (cluster 2), myeloid dendritic cells (cluster 3), PT epithelial cells (cluster 4), CD8+ effector cells (cluster 5), basal cells (cluster 6), and epithelial cells (cluster 7; Figs. 4E and 4F). Noteworthy is the presence of PTCs in the urine sample, although these urine PTCs exhibited high expression of mitochondrial genes, such as MT1L, MT1M, MTCO3P12, and MTND2P28, indicating their pre-apoptotic state.

In addition to mitochondrial genes, the expression of CD52, SERPINA1, PLAU, GLTP, and SAFB2 also exhibited significant increases (Table S5). CD52 can induce T-cell activation by direct binding to the T-cell receptor. A humanized anti-CD52 monoclonal antibody, Alemtuzumab, has been approved for the treatment of relapsing multiple sclerosis (Li et al., 2018; Zhao et al., 2017), suggesting its potential benefit in nephritis. The increase in SERPINA1 has been associated with acute inflammatory conditions, and urine SERPINA1 was associated with proteinuria (Starodubtseva et al., 2020), indicating its potential as a diagnostic marker.

DEGs of PTCs in urine and PBMC are depicted in Fig. 4G. The GO analysis of these DEGs highlighted that the top terms in biological process (BP) were related to immune activation, particularly targeting neutrophils and interferon (IFN)- *γ*, such as neutrophil activation and response to infection (Fig. 4H). Additionally, cell adhesion and Golgi-endoplasmic reticulum were major terms within the cellular component (CC) category. Cell adhesion plays a crucial role in PTC detachment, while the Golgi-endoplasmic reticulum may participate in major histocompatibility complex (MHC) expression. This finding is supported by the molecular function (MF) category where adhesion-related pathways and antigen-presenting pathways were prominent. Hence, it can be concluded that pathological changes lead to a decrease in cell adhesion ability and induce inflammation, ultimately resulting in PTC exfoliation and apoptosis.

### Upregulated inflammation of PTCs and neutrophils in the kidney

**Figure 5:**
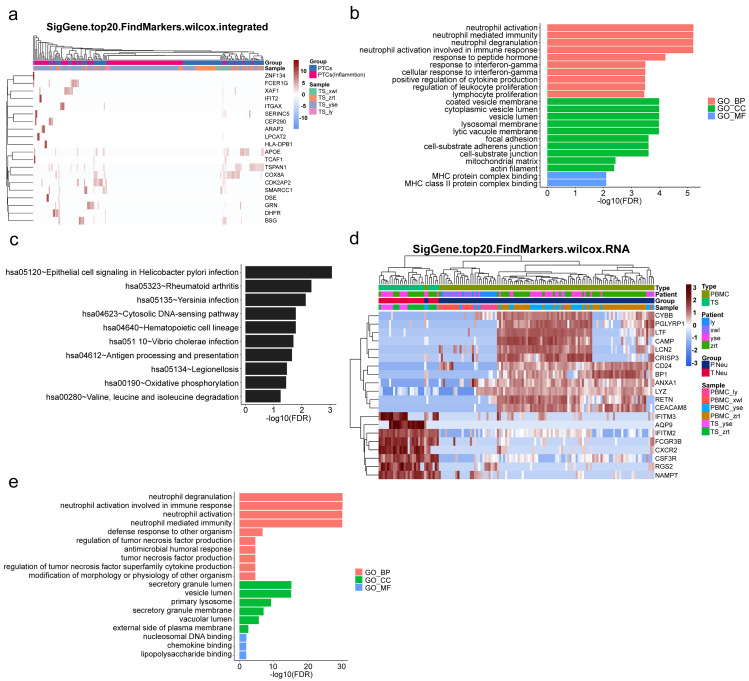
Upregulated inflammation in the proximal tubule cells (PTCs) and neutrophils of the kidney. (A) Top 20 differentially expressed genes (DEGs) of PTCs and Inflam-PTCs. (B) Gene Ontology (GO) analysis of DEGs of PTCs and Inflam-PTCs. (C) Top 10 significantly enriched pathways of DEGs. (D) Top 20 DEGs in neutrophils derived from the tissues and peripheral blood mononuclear cells (PBMCs). (E) GO analysis of DEGs of neutrophils derived from the tissue and PBMCs.

To gain further insight into the mechanisms underlying kidney pathological changes, DEGs in PTC and Inflam-PTC were subjected to further analysis. A total of 448 DEGs with fold-change greater than 2 or less than 0.5 were identified. The heatmap of the top 20 DEGs in the two clusters is shown in Fig. 5A (Table S6), including ZNF134, TSPAN1, and IFIT2, among others. Enrichment analysis revealed 58 GO terms, with the majority being associated with BP (45 terms), followed by 11 terms related to CC, and two terms linked to MF.

As depicted in Fig. 5B, all the leading terms within the BP category are associated with immune activation, particularly focusing on neutrophil and IFN-r activities. These activities include neutrophil activation, neutrophil-mediated immunity, neutrophil degranulation, neutrophil activation in the immune response, and cellular response to IFN-r. Other noted processes involve the positive regulation of cytokine production and lymphocyte proliferation. These processes are central to the enhancement of inflammation and could also serve as therapeutic targets for kidney diseases. Analyzing CCs offers insight into why inflammatory PTCs might detach from tissue and enter the blood and urine. Many terms pertain to cell–cell interaction, including cell adhesion and junctions. Pathological alterations in these components could weaken cell-to-cell and cell–matrix interactions, leading to exfoliation. Only two terms are related to molecular function, both of which concern MHC protein binding. This is in line with previous findings that MHC II expression increases during inflammation, driven by the class II transactivator (CIITA) commonly induced by IFN- *γ* (Heuberger, Pott & Maloy, 2021). The prominent presence of IFN- *γ* in the top BP supports this MF.

KEGG analysis indicates that DEGs are segmented into 288 pathways (as shown in Fig. 5C, Table S7). Half of the top ten enriched pathways are associated with infections (including epithelial cell signaling in *Helicobacter pylori* infection, *Yersinia* infection, *Vibrio cholerae* infection, legionellosis caused by *Legionella*, and antigen processing and presentation). This points to inflammatory changes in the kidney. Moreover, DEGs are also heavily present in rheumatoid arthritis, which has fibrosis and lesion mechanisms akin to nephropathy.

As pathways linked to neutrophil activation consistently emerge in our analyses, we further studied DEGs in neutrophils derived from tissue and PBMCs. A total of 131 DEGs with significant fold changes (>2 or <0.5) were identified (Table S8). Figure 5D highlights the top 20 DEGs. Tissue-resident neutrophils show high expression levels of several genes, including IFITM3, AQP9, IFITM2, FCGR3B, CXCR2, CSF3R, RGS2, and NAMPT. The genes IFITM2 and IFITM3 showed upregulated expression in urine PTCs, which is involved in inflammation. Notably, CXCR2, an interleukin (IL)-8 receptor, plays a key role in numerous inflammatory diseases through various, including the PI3K, p38/ERK, and JAK pathways. Its upregulation boosts the migration and infiltration of neutrophils, promoting chronic inflammation (An et al., 2019; Cheng et al., 2019). In this study, increased CXCR2 in neutrophils correlated with heightened kidney neutrophil infiltration, worsening the nephritis condition. Additionally, AQP9 is essential for regulating water and salt homeostasis in human polymorphonuclear leukocytes and influences neutrophil motility, polarization, and chemotaxis of neutrophils (Karlsson et al., 2011). Patients with systemic inflammatory response syndrome showed increased AQP9 expression in neutrophils compared with healthy controls (Matsushima et al., 2014). The result indicated that kidney-resident neutrophils are activated and may drive inflammatory responses in the kidney.

GO analysis reveals 79 terms, comprising 70 for biological processes, six for CCs, and three for molecular functions (as illustrated in Fig. 5E, Table S9). Top BP terms predominantly relate to neutrophil activation and immune response, including activities such as neutrophil degranulation and tumor necrosis factor production. CC terms touch upon degranulation and migration, while MF terms such as nucleosomal DNA binding, chemokine binding, and lipopolysaccharide binding are linked to neutrophil activation. Collectively, the GO terms emphasize the involvement of neutrophil activation and immune functions, underscoring the kidney’s inflammatory condition.

### Inflammatory process of PTCs

**Figure 6:**
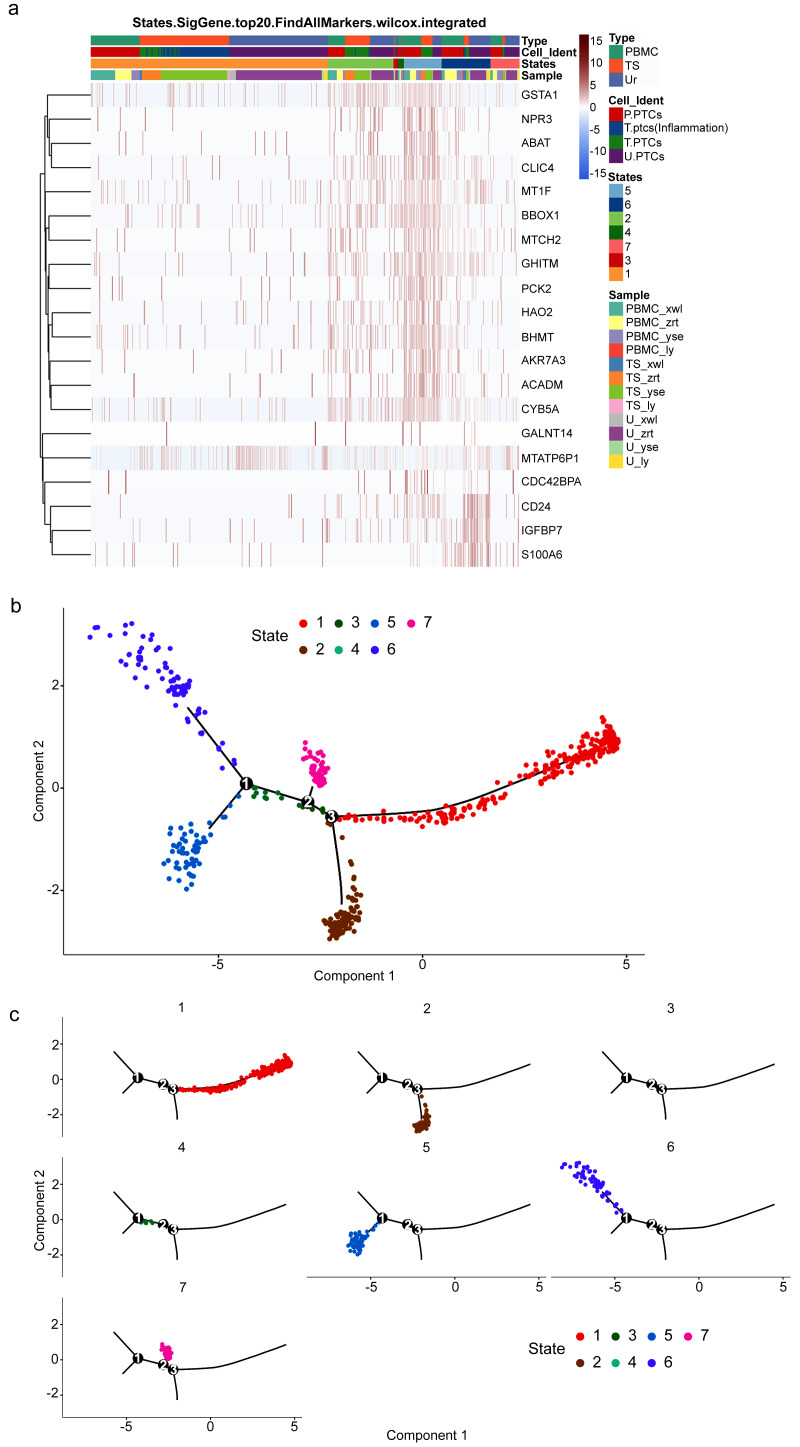
Potential developmental trajectories of proximal tubule cells (PTCs) based on their expression of marker genes. (A) Top 20 markers of PTCs. (B) Branched structure of the CD8+ T-cell developmental trajectory in each patient. (C) Distribution of PTCs in each statue.

To gain insight into the developmental shifts occurring during nephritis, we utilized the unsupervised inference method Monocle to map potential developmental trajectories of PTCs (Qiu et al., 2017). The top 20 markers of cells in the seven statues of different samples are shown in Fig. 6A. The statue of PTCs was determined according to their gene expression patterns (Table S10).

Cells of statue 1 have high expression of IL1B and inflammatory gene calmodulin 3. IL1B has been recognized as a marker for cellular stress, contributing not only to anti-infection activities but also to various inflammatory-associated disorders and acute tissue damage (Lopez-Castejon & Brough, 2011). Thus, cells of statue 1 were classified as pro-inflammatory PTCs.

Nitrilase 2 (Nit2) is responsible for catalyzing the hydrolysis of *α*-ketosuccinamate into oxaloacetate. Overexpression of Nit2 has been shown to arrest cells in the G2 phase by inhibiting 14-3-3 *β* expression and enhancing 14-3-3 *σ* expression (Lin et al., 2007). Another highly expressed gene in this cluster is GSTA1, which encodes an enzyme responsible for adding glutathione to electrophilic compounds, including therapeutic drugs, toxins, and products of oxidative stress. GSTA1 can suppress the activation of JNK signaling in response to pro-inflammatory and oxidative stress, thereby protecting cells from JNK-associated apoptosis (Romero et al., 2006). Accordingly, Statue 2 can be classified as acute injured PTCs with cell cycle arrest.

TMEM106B regulates lysosomal function, including lysosome pH, exocytosis, and trafficking. Lysosomes play a protective role against kidney injury, and PTCs with high expression of TMEM106B may indicate a pro-apoptotic state (Feng et al., 2020). Therefore, PCTs in Statue 3 can be classified as pro-apoptotic PCTs.

ZC3HAV1 is a member of the PARP proteins-enzymes family. It not only promotes the migration and invasion of PC cells but also induces epithelial-mesenchymal transition (Huang et al., 2021). Furthermore, Cathepsin W (CTSW) is highly expressed in cells of Statue 4. Its expression is commonly found in blood cells, especially in immune cells (Raab et al., 2011). Thus, PTCs in Statue 4 are circulating PTCs.

PTCs in Statue 5 exhibit high expression of genes involved in energy production, such as ACADM, AKR7A3, and HAO2. ACADM catalyzes the first dehydrogenation step in the oxidation of fatty acyl-CoA esters in mitochondria, and HAO2 catalyzes the oxidation of long-chain 2-hydroxyl acidic substrates (Bloom et al., 2018; Xiao et al., 2019). Therefore, PTCs in Statue 5 are functionally normal PTCs.

IGFBP7 is a cell-cycle arrest protein expressed in renal tubular cells during periods of cellular stress or injury. Urine IGFBP7 has been approved as a marker by the US Food and Drug Administration for determining the risk of developing moderate-to-severe acute kidney injury (Vijayan et al., 2016), indicating that PTCs in Statue 6 are injured and inflammatory PTCs.

PTCs in Statue 7 have high expression of MATLAT1. MATLAT1 is widely expressed in vascular endothelial cells and plays an important role in regulating vascular growth. Silencing of MATLAT1 significantly reduces the proliferation of endothelial cells, inhibits vascularization, and decreases capillary density (Michalik et al., 2014). It is also involved in the protection of cerebral microvessels after ischemic insult, and silencing of MATLAT1 in brain microvascular endothelial cells increases the expression of pro-apoptotic factors and proinflammatory cytokines (Zhang et al., 2016; Zhang et al., 2017). Thus, MATLAT1 may play an important role in anti-inflammatory and wound-healing processes. Hence, PTCs in Statue 7 are cells in a state of wound healing or functional restoration.

Consequently, a branched structure of PTCs developmental trajectory is shown in Fig. 6B, beginning with functionally normal PTCs (Statue 5), progressing through an intermediate injured and recovery state (acute injured PTCs (Statue 2), injured and inflammatory PTCs (Statue 6), wound-healing PTCs (Statue 7)), followed by the pro-inflammatory PTCs (Statue 1), and ending with pro-apoptotic PCTs (Statue 3) and detached PCTs (circulating PTCs in Statue 4).

The distribution of PTCs in each statue is shown in Fig. 6C. Pro-inflammatory PTCs account for the largest number of PTCs, followed by injured and inflammatory PTCs in Statue 6. The cell distribution is consistent with the pathology observed in the four patients, who were confirmed as having acute or chronic nephritis. PTCs developmental trajectory indicates that inflammation may be the initiator of nephritis, and results in the apoptosis of PCTs. Therefore, anti-inflammation and anti-apoptotic drugs may relive the course of disease.

## Discussion

In this study, we conducted single-cell transcriptomics of tissue, PBMC, and urine from patients with MN, IN, PSG, and DN. This unveiled a diversity of cell subtypes and subtype-specific gene dysregulation in nephritis, which may serve as clinical indicators.

Our most significant finding is that PTCs from patients with nephritis can migrate into both the circulatory system and urine. Contrastingly, no PTCs were identified in healthy PBMCs. Thus, analyzing the gene profiles of these migrating PTCs could offer early, insightful diagnostic clues and evaluations of nephritis progression. As more nephritis samples are studied, gene typing of these PTCs might present a less invasive alternative to renal biopsy. Intriguingly, the developmental trajectories of PTCs in nephritis outline the pathological stages, especially the acute injured PTCs (Statue 2), inflammatory PTCs (Statue 6), wound-healing PTCs (Statue 7), and pro-apoptotic PTCs (Statue 3). Notably, acutely injured PTC is a key marker for early and fast diagnosis of acute nephritis. The percentage of various PTC states may also aid prognosis assessment.

Furthermore, within a single cell type, both functionally normal and abnormal cells can exist. For instance, alongside regular fibroblast cells, pro-fibrosis fibroblast cells, mesangial cells, and pro-apoptotic mesangial cells, PTCs and inflammatory PTCs in cluster 0 display elevated LTA4H expression, which can enhance neutrophil and T-cell influx through LTB4 (Wan et al., 2017). This aligns with our GO analysis that highlights activated neutrophil and IFN-r-related pathways. Continuous immune cell infiltration can induce sterile inflammation and chronic inflammatory diseases (Subramanian, Majumdar & Parent, 2017). This suggests that pro-fibrosis fibroblast cells play an important role in nephritis inflammation and progression, which can be a potential target for the disease.

Our GO analysis of DEGs of different cell groups such as PTCs in PBMCs and tissue, inflammatory PTCs and PTCs, as well as urine and tissue revealed that top terms primarily pertain to neutrophil chemotaxis, activation, IFN-r signaling, and reduced cell adhesion. Notable inflammatory changes were detected in PTCs and their adjacent tissue in our MN, IN, DN, and PSG patient samples. This indicates that unchecked PTC inflammation can initiate chemotaxis and activate neutrophils, causing them to detach from their resident sites and enter the bloodstream; thus, it serves as a promising diagnostic biomarker for nephropathy. These biological processes are central to inflammation enhancement, potentially offering therapeutic targets for kidney disease.

Compared with PBMC-derived neutrophils, tissue-resident neutrophils exhibited higher pro-inflammatory. The genes IFITM3, AQP9, IFITM2, CSF3R and CXCR2. IFITM2 and IFITM3 showed up-regulation in urine PTCs, suggesting their potential as nephritis markers. The IL-8 receptor CXCR2 is instrumental in various inflammatory diseases, orchestrating multiple pathways such as PI3K, p38/ERK, and JAK (Cheng et al., 2019). This receptor is crucial for the chemotaxis and activation of neutrophils in the kidney. Notably, in a murine model with LPS-induced neuroinflammation, a CXCR2 antagonist was shown to significantly reduce neutrophil infiltration into the brain (Wu et al., 2020). This suggests that inhibiting IL-8 signaling could offer therapeutic advantages in managing nephritis inflammation. Additionally, our GO analysis of DEGs highlighted that most of the primary GO terms relate to neutrophil migration, activation, and immune response. Hence, it is evident that neutrophils play an integral role in the onset and progression of nephritis and may serve as a promising therapeutic target.

The scRNA-seq analysis results suggest that the chemotaxis and activation of neutrophils may precede nephritis, eventually causing sterile and chronic inflammation of PTCs. Pro-fibrosis fibroblast cells and the IFITM signaling pathways are key factors in nephritis pathology. Future drug development might focus on modulating these inflammatory pathways. Moreover, with more diverse nephritis samples, we anticipate the identification of precise biomarkers or gene expression patterns for each nephritis type. Consequently, urine PTC testing might emerge as a noninvasive early diagnostic tool for nephritis.

## Conclusion

In summary, our research at the single-cell level elucidates the pathological shifts in renal cells of nephritis patients, unraveling the immune mechanisms propelling disease progression. The inflammatory effects of PTCs and fibroblasts on neutrophils are fundamental to nephritis’ evolution. Additionally, a noninvasive urine PTC test could enhance early nephritis diagnosis and treatment. Nevertheless, to authenticate our findings, further studies encompassing diverse nephritis types and larger sample sizes are imperative.

##  Supplemental Information

10.7717/peerj.16499/supp-1 Supplemental Information 1 Supplementary tables and raw data

## Additional Information and Declarations

### DNA Deposition

The following information was supplied regarding the deposition of DNA sequences: The sequences are available at GEO: GSE203406.

### Data Availability

The following information was supplied regarding data availability: The raw data is available in the Supplementary Files.
