# Supplementary figures and images for: Proximal tubule cells in blood and urine as potential biomarkers for kidney disease biopsy
Source: PeerJ. 2023 Dec 6;11:e16499. doi: 10.7717/peerj.16499 (PMC10710128; doi:10.7717/peerj.16499)

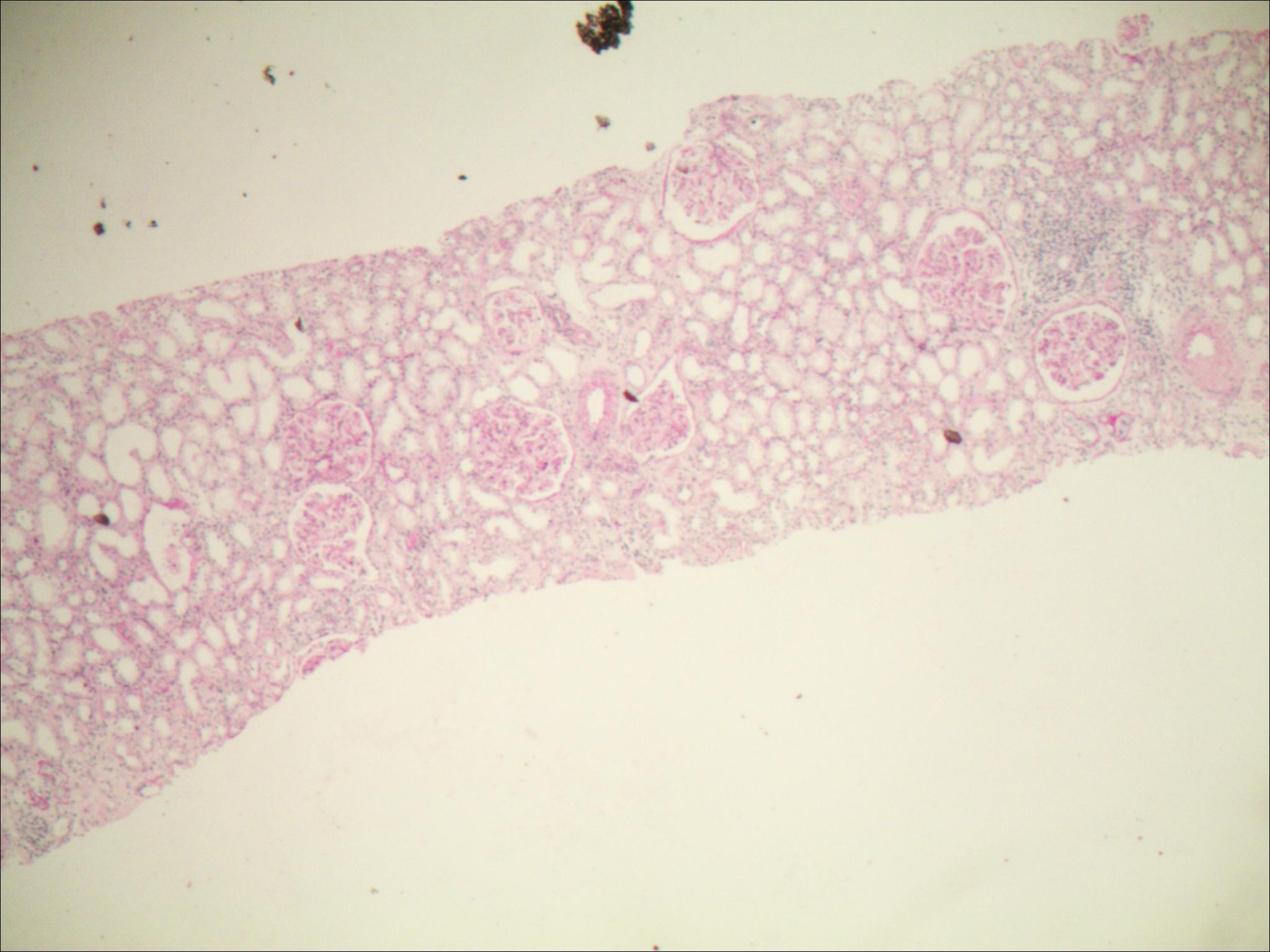

Supplement: Supplemental Information 1 [file peerj-11-16499-s001.zip › Supplementary Materials/Raw data/figure1a/luo_2.jpg]

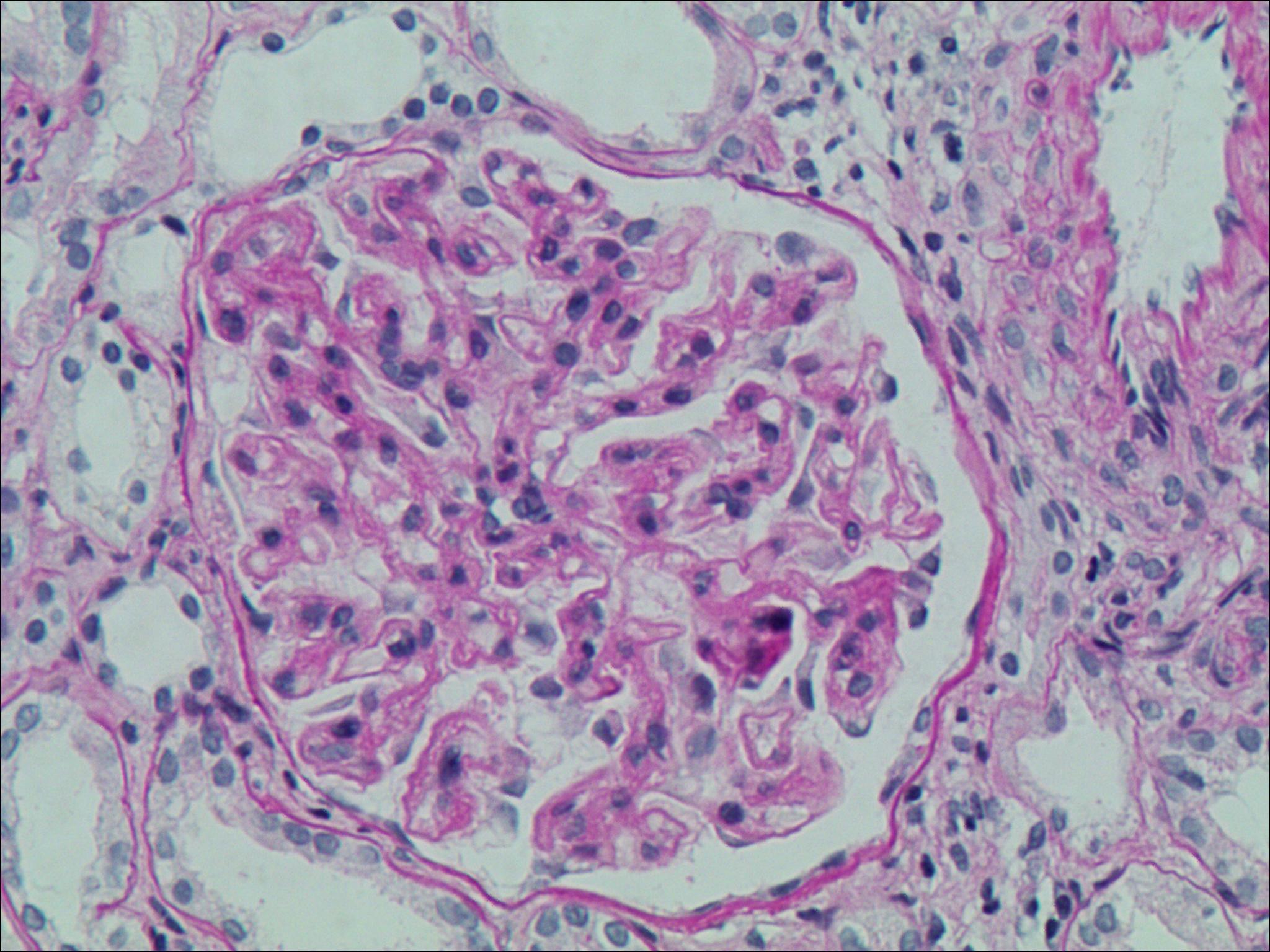

Supplement: Supplemental Information 1 [file peerj-11-16499-s001.zip › Supplementary Materials/Raw data/figure1a/luo_3.jpg]

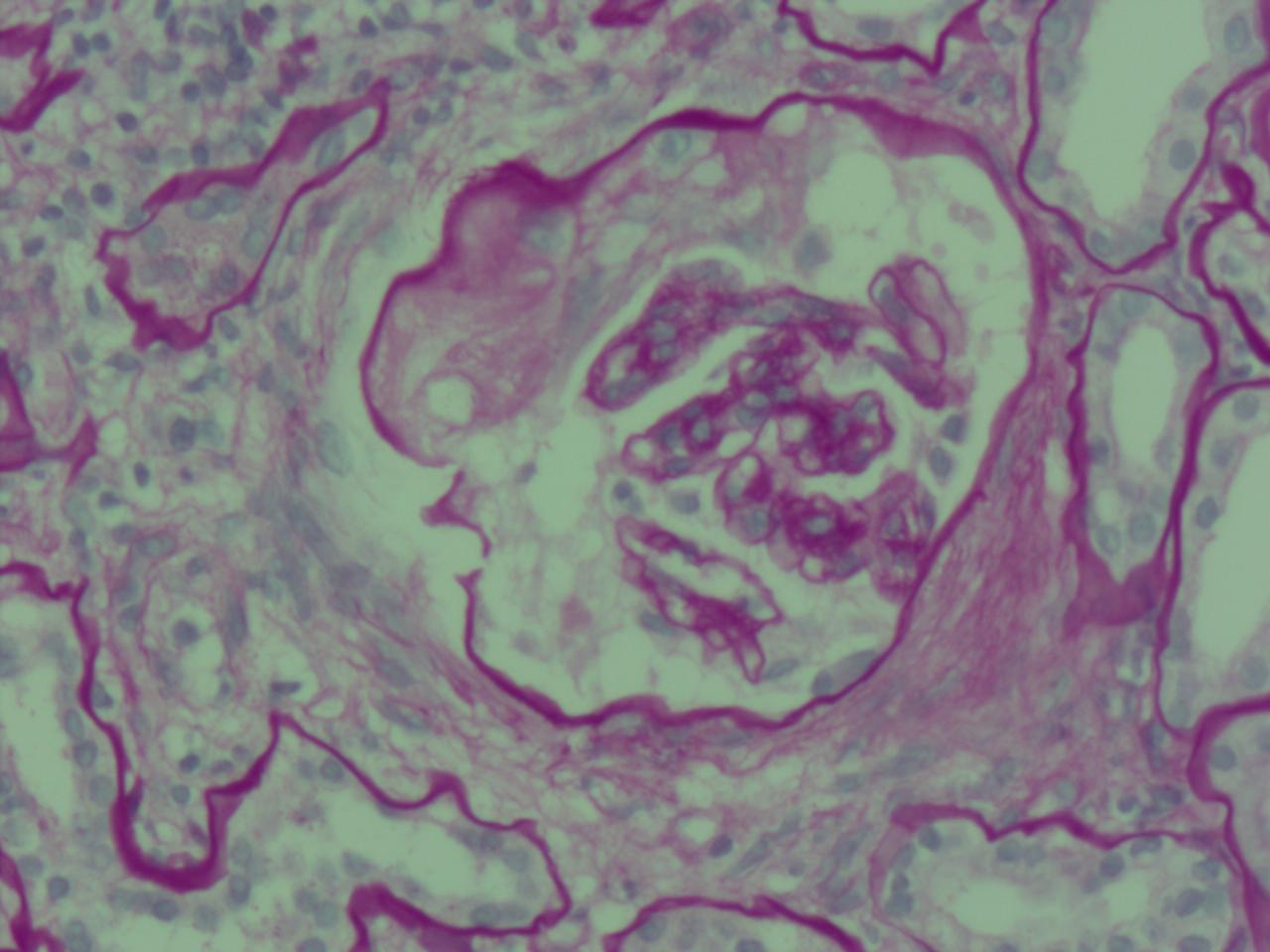

Supplement: Supplemental Information 1 [file peerj-11-16499-s001.zip › Supplementary Materials/Raw data/figure1a/xie_2.jpg]

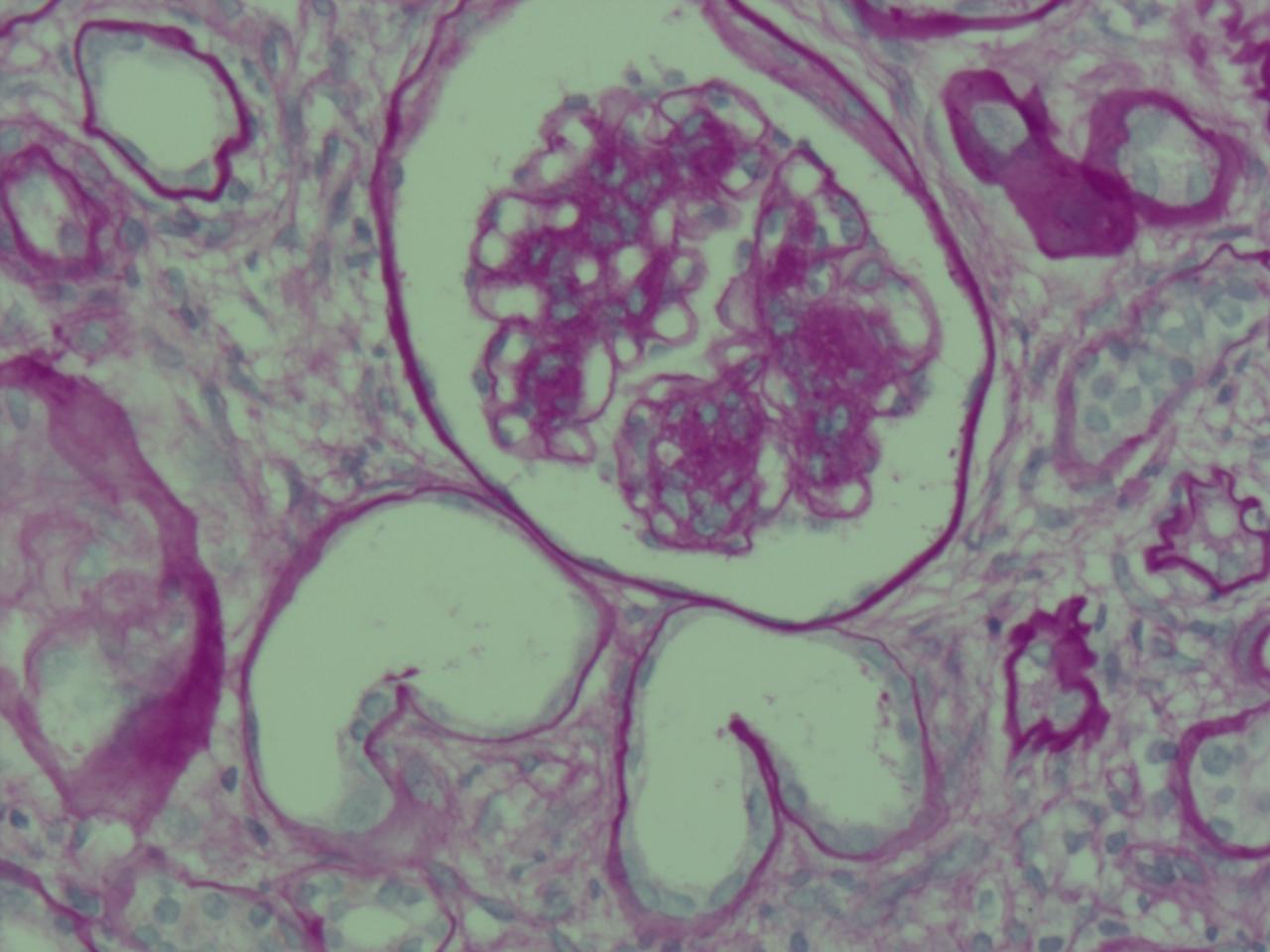

Supplement: Supplemental Information 1 [file peerj-11-16499-s001.zip › Supplementary Materials/Raw data/figure1a/xie_3.jpg]

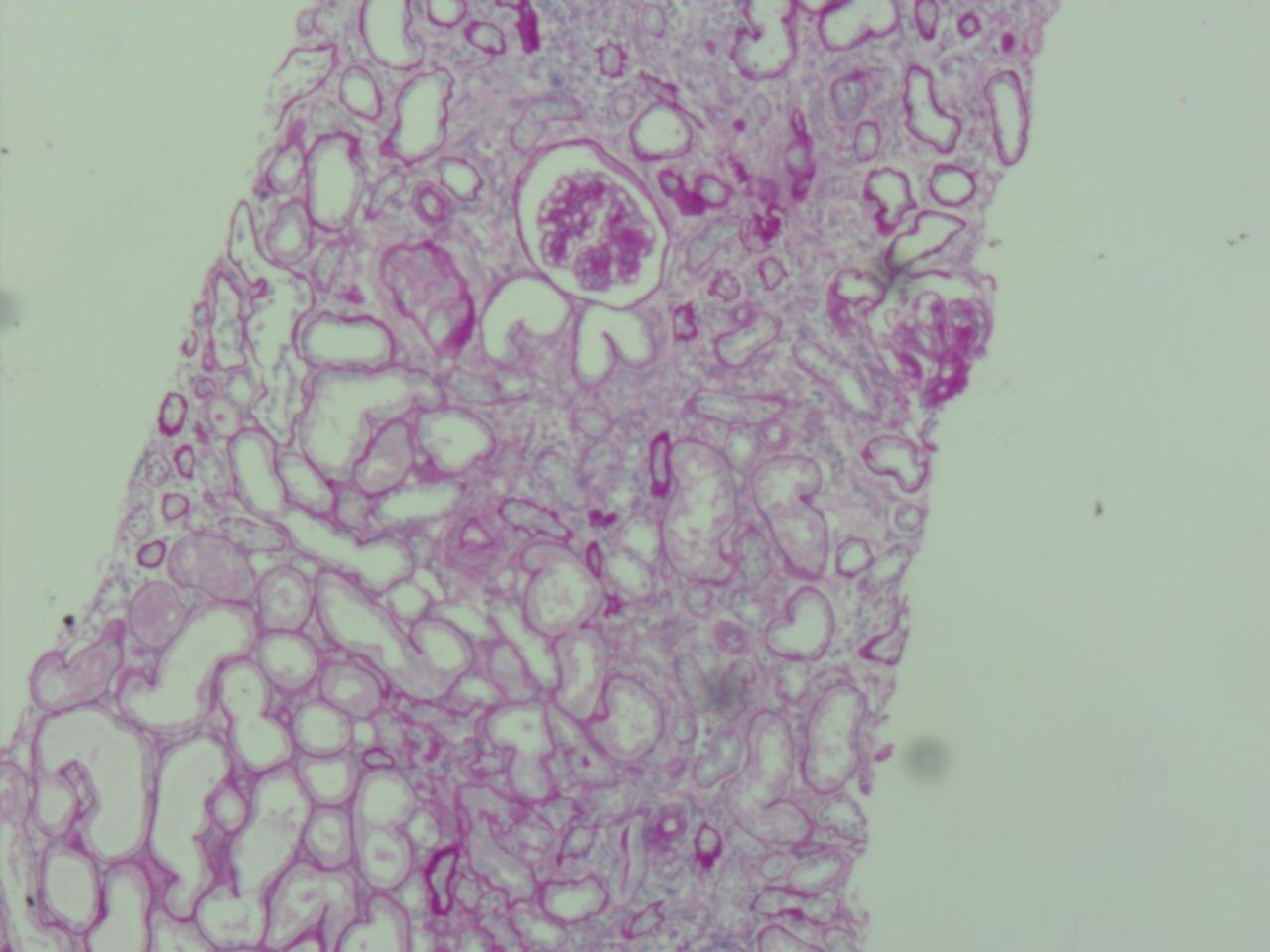

Supplement: Supplemental Information 1 [file peerj-11-16499-s001.zip › Supplementary Materials/Raw data/figure1a/xie_4.jpg]

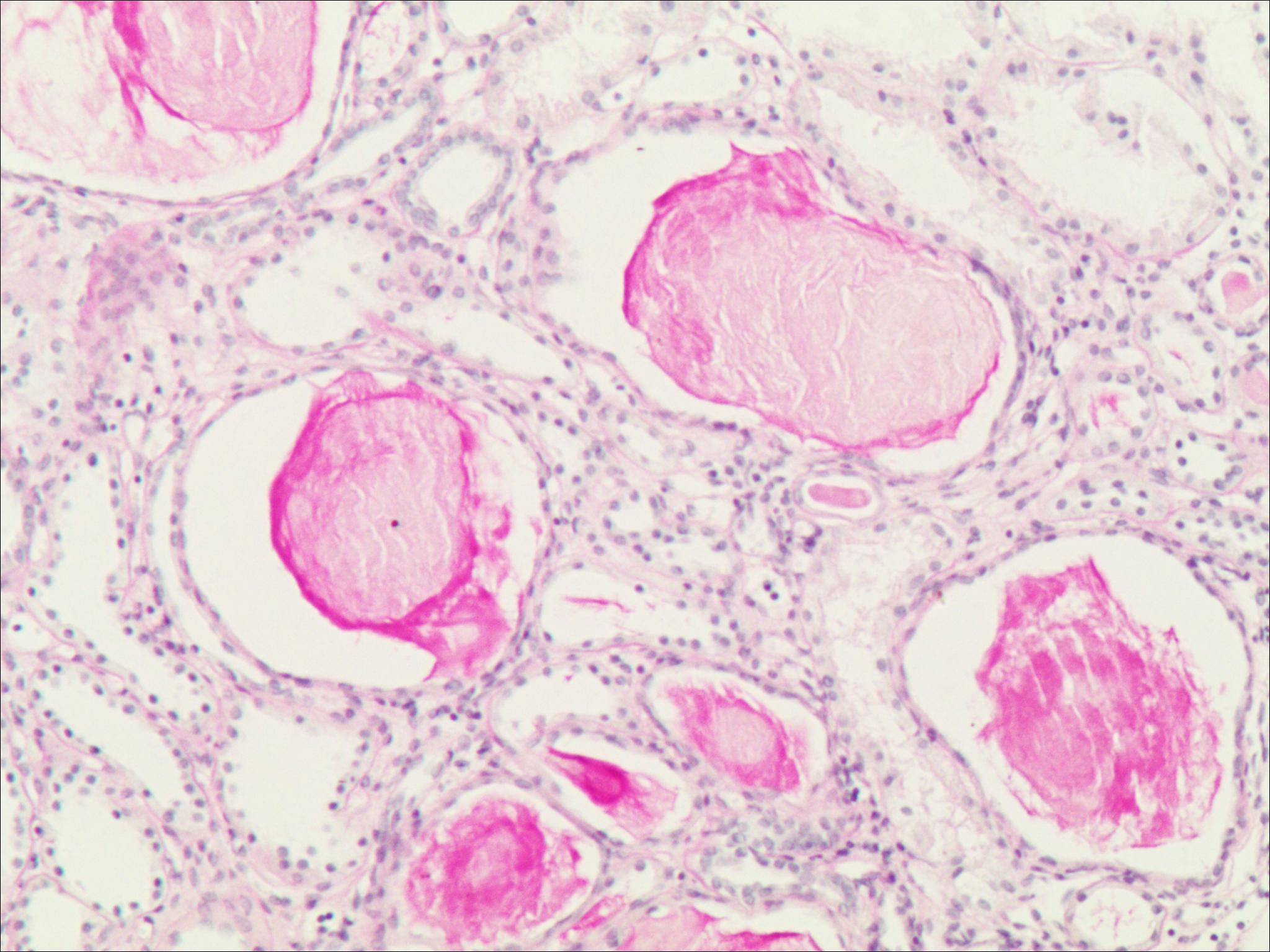

Supplement: Supplemental Information 1 [file peerj-11-16499-s001.zip › Supplementary Materials/Raw data/figure1a/yi_2.jpg]

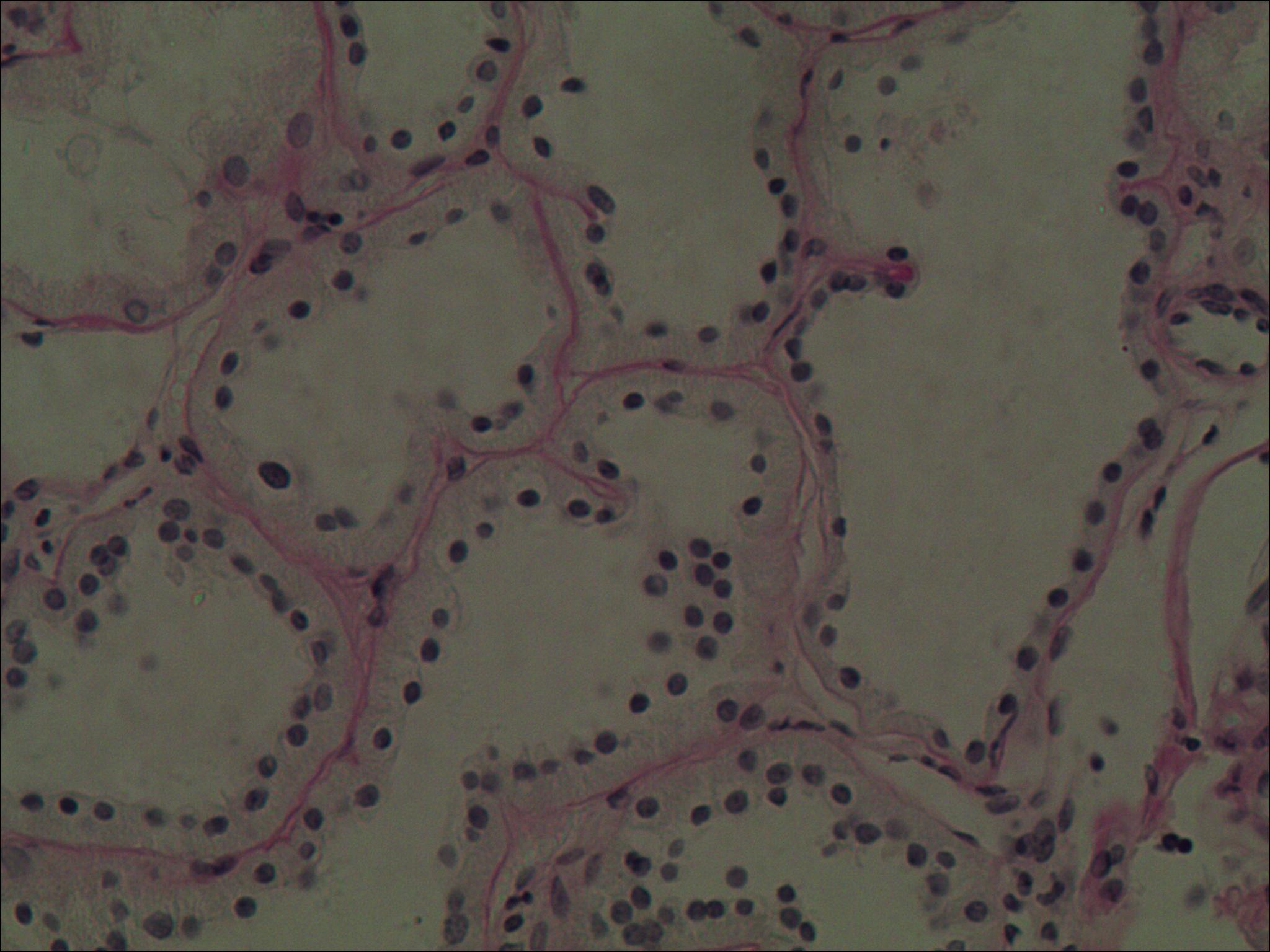

Supplement: Supplemental Information 1 [file peerj-11-16499-s001.zip › Supplementary Materials/Raw data/figure1a/yi-3.jpg]

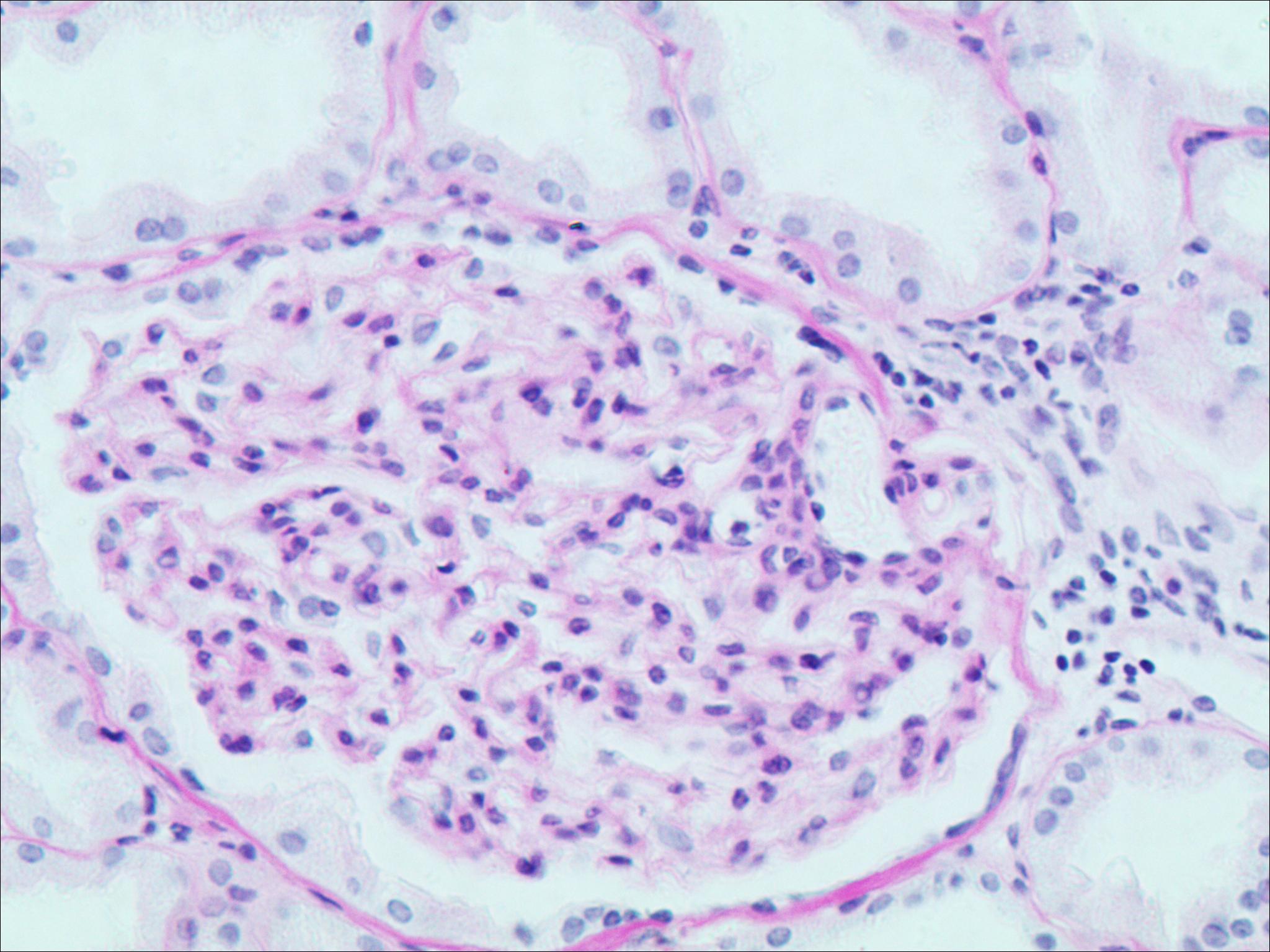

Supplement: Supplemental Information 1 [file peerj-11-16499-s001.zip › Supplementary Materials/Raw data/figure1a/yi_5.jpg]

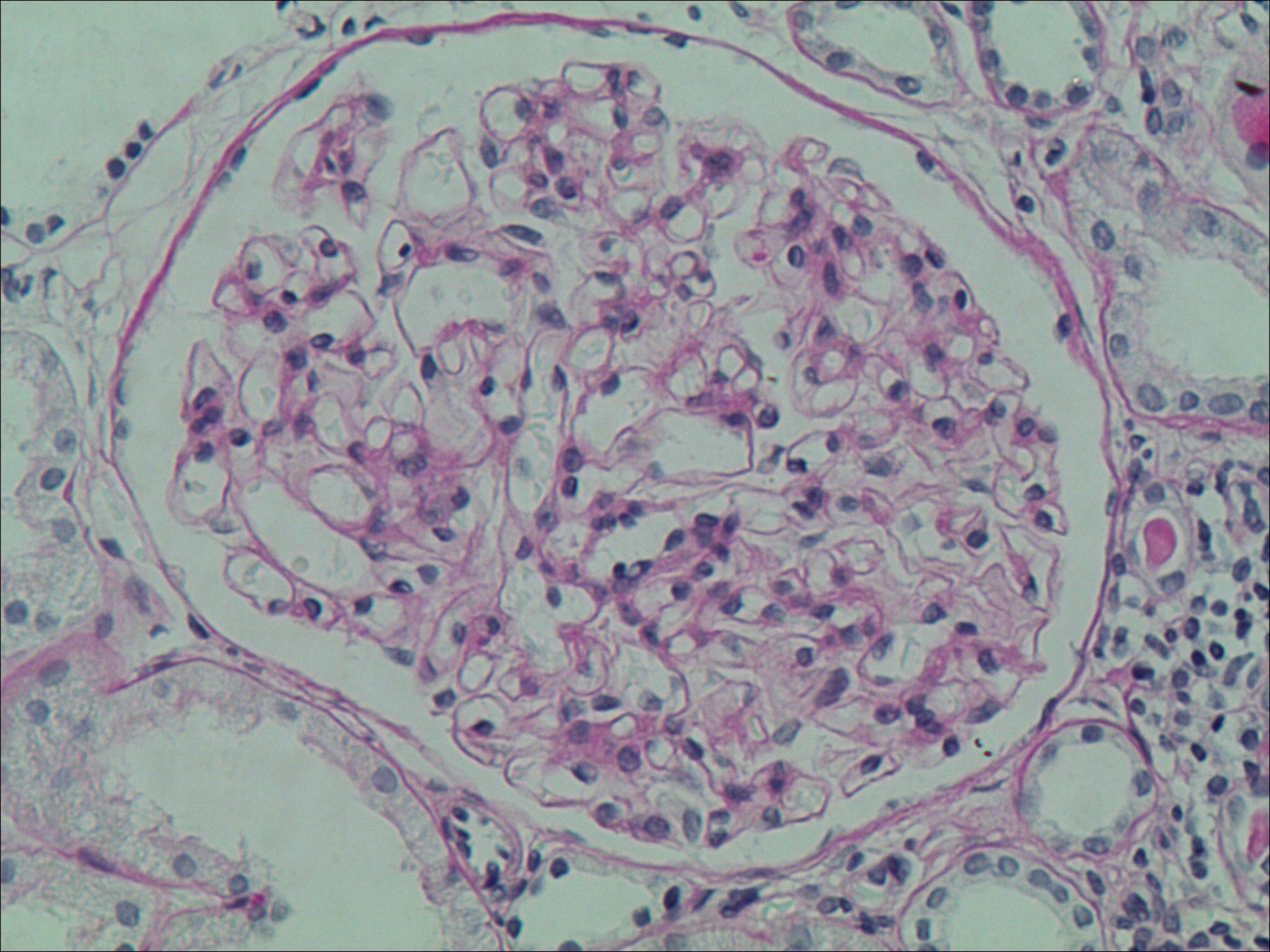

Supplement: Supplemental Information 1 [file peerj-11-16499-s001.zip › Supplementary Materials/Raw data/figure1a/zhang_2.jpg]

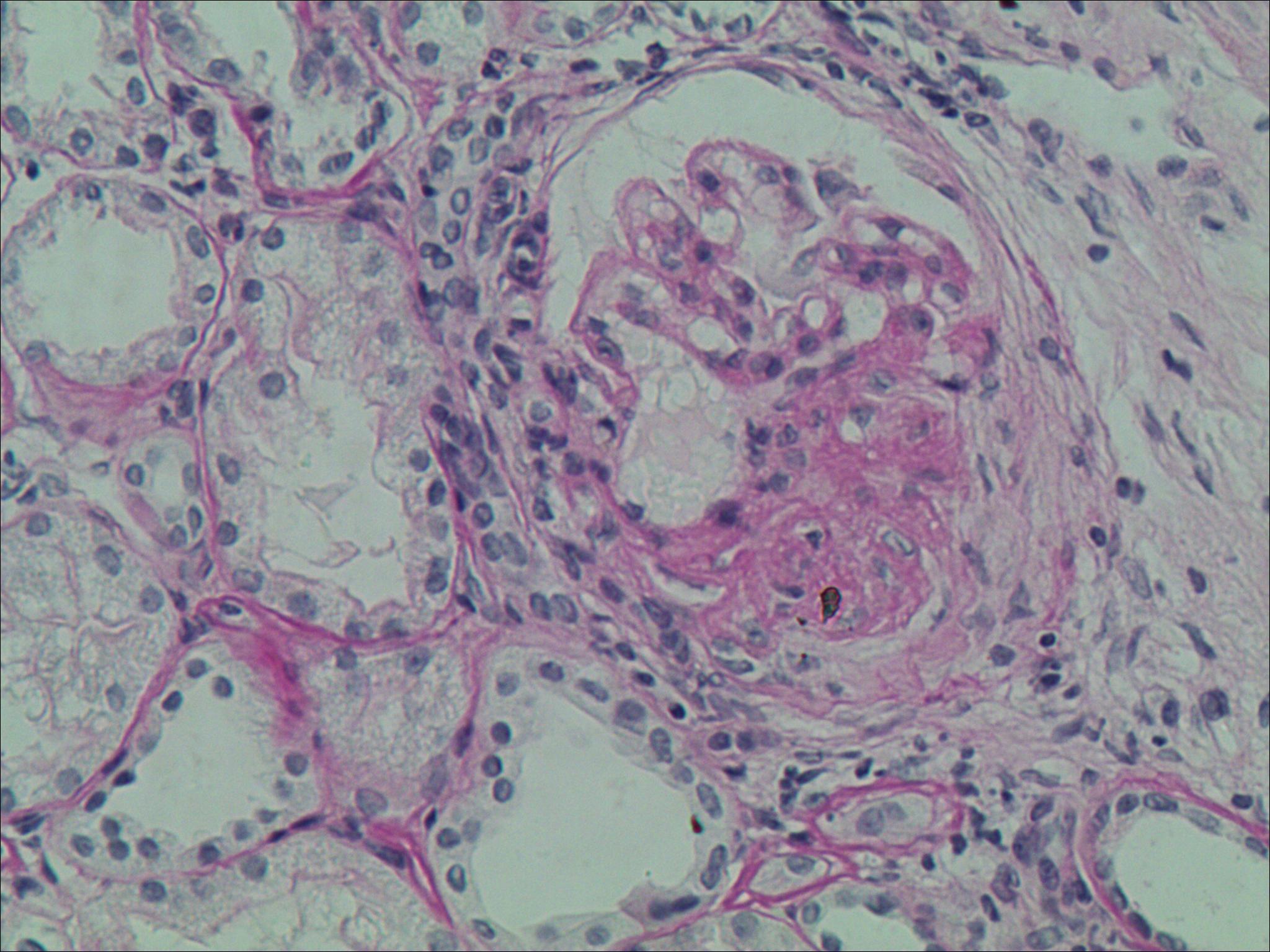

Supplement: Supplemental Information 1 [file peerj-11-16499-s001.zip › Supplementary Materials/Raw data/figure1a/zhang_3.jpg]

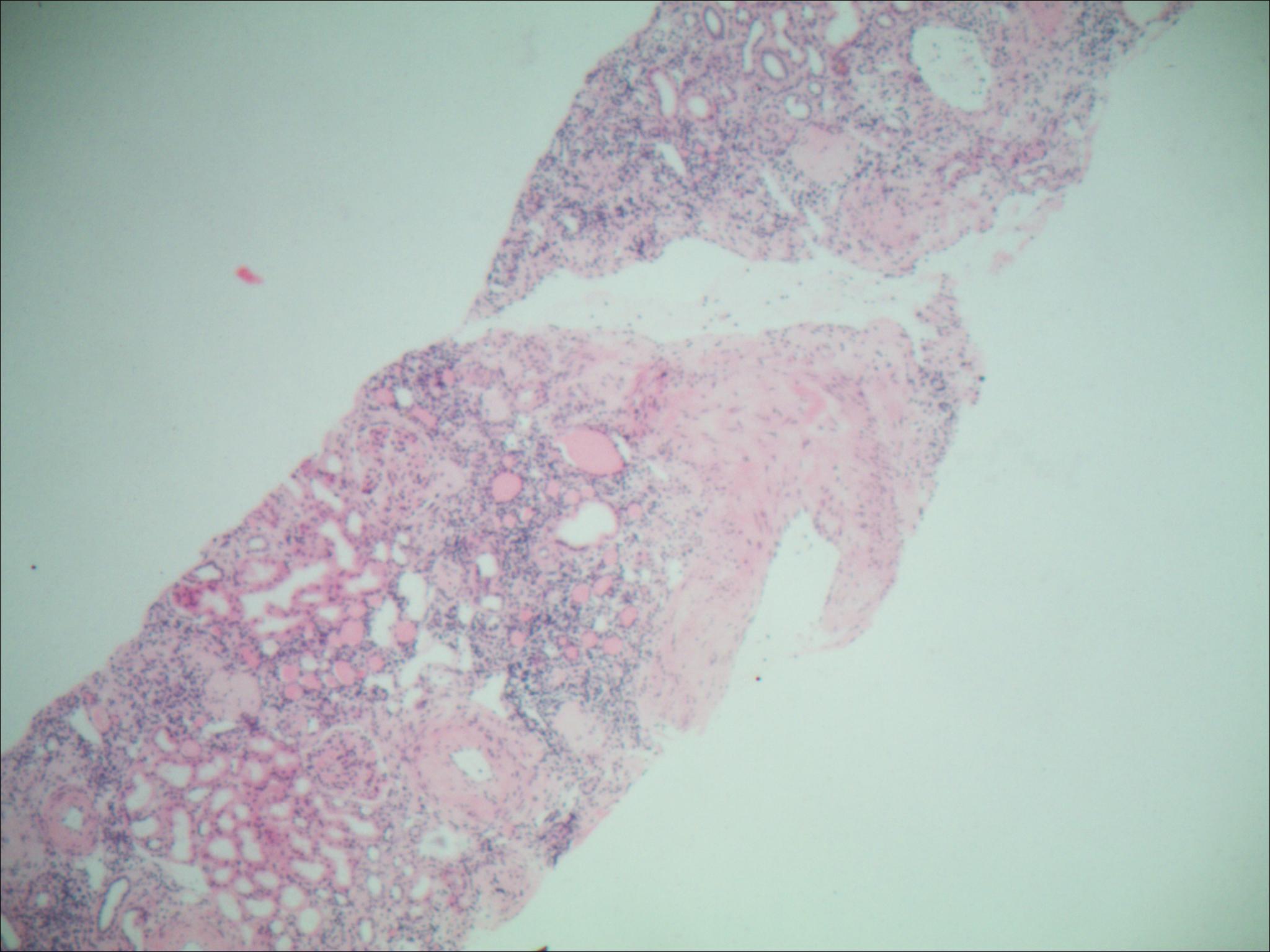

Supplement: Supplemental Information 1 [file peerj-11-16499-s001.zip › Supplementary Materials/Raw data/figure1a/zhang_4.jpg]
